# Reply to: Exercise Testing and Artificial Intelligence as Allies in Improving the Detection and Diagnosis of Long QT Syndrome

**DOI:** 10.1016/j.mcpdig.2024.01.012

**Published:** 2024-03-01

**Authors:** Negar Raissi Dehkordi, Nastaran Raissi Dehkordi, Kimia Karimi Toudeshki, Mohammad Hadi Farjoo

**Affiliations:** Department of Pharmacology, Shahid Beheshti University of Medical Sciences, Tehran, Iran

To the Editor:

We thank Dr. Audrey Harvey et al for their Letter to the Editor in response to our previously published article about artificial intelligence in the diagnosis of long QT syndrome (LQTS).^1^ Their emphasis on the crucial link between LQTS and exercise testing aligns well with our understanding of the diagnostic challenges posed by the corrected QT interval range overlap between LQTS and healthy individuals. Exercise testing is instrumental in identifying and evaluating patients at risk of LQTS, especially those with concealed QT interval prolongation. Their proposal to integrate resting electrocardiogram data with exercise electrocardiogram data for artificial intelligence optimization provides an intriguing opportunity to improve the accuracy of artificial intelligence models in LQTS diagnosis and address the limitations we discussed in our review, specifically concerning the potential misclassification of healthy controls.

## Potential Competing Interests

The authors report no competing interests.
